# Identification of the selected soil bacteria genera based on their geometric and dispersion features

**DOI:** 10.1371/journal.pone.0293362

**Published:** 2023-10-27

**Authors:** Aleksandra Konopka, Ryszard Kozera, Lidia Sas-Paszt, Pawel Trzcinski, Anna Lisek

**Affiliations:** 1 Institute of Information Technology, Warsaw University of Life Sciences - SGGW, Warsaw, Poland; 2 School of Physics, Mathematics and Computing, The University of Western Australia, Perth, Australia; 3 Department of Microbiology and Rizosphere, The National Institute of Horticultural Research, Skierniewice, Poland; University of A Coruña, SPAIN

## Abstract

The visual analysis of microscopic images is often used for soil bacteria recognition in microbiology. Such task can be automated with the aid of machine learning and digital image processing techniques. The best results for soil microorganism identification usually rely on extracting features based on color. However, accommodating in the latter an extra impact of lighting conditions or sample’s preparation on classification accuracy is often omitted. In contrast, this research examines features which are insensitive to the above two factors by focusing rather on bacteria shape and their specific group dispersion. In doing so, the calculation of layout features resorts to *k*-means and mean shift methods. Additionally, the dependencies between specific distances determined from bacteria cells and the curvature of interpolated bacteria boundary are computed to extract vital geometric shape information. The proposed bacteria recognition tool involves testing four different classification methods for which the parameters are iteratively adjusted. The results obtained here for five selected soil bacteria genera: *Enterobacter*, *Rhizobium*, *Pantoea*, *Bradyrhizobium* and *Pseudomonas* reach 85.14% classification accuracy upon combining both geometric and dispersion features. The latter forms a promising result as a substitutive tool for color-based feature classification.

## Introduction

Identification of bacteria can be realized with the use of many molecular techniques, including ribotyping, repetitive extragenic palindromic PCR (Rep-PCR), denaturing gradient gel electrophoresis (DGGE), terminal (T)-restriction fragment length polymorphism (T-RFLP), multilocus sequence typing (MLST) and whole-genome sequencing (WGS) [1]. MLST uses DNA sequencing of internal fragments of the housekeeping gene loci (seven in number) of bacterial strains to characterize alleles [2]. In practice, a common stance for bacteria identification is based on sequence analysis of 16 SrRNA gene [3, 4] and MLST unveiling the same intraspecific genetic structure patterns as genomes [5]. In bacteria recognition process, the morphological features can also be considered while analyzing the microscopic images. However, sometimes it is hard to distinguish between different bacteria species due to their morphological similarities within a genera [6]. The image-based identification can be tedious and laborious.

**Fig 1:**
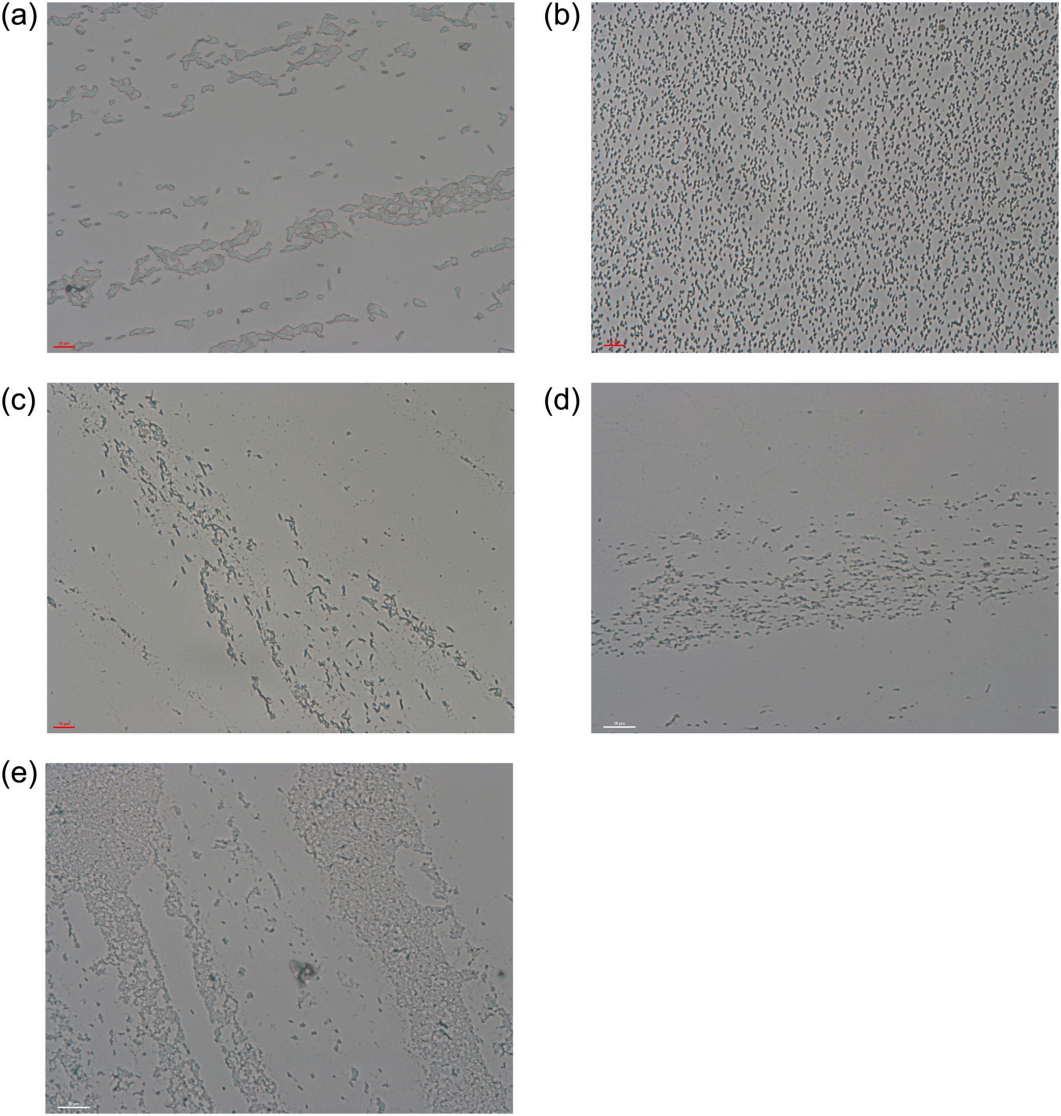
Examples of bacteria images: (a) *Enterobacter*, (b) *Rhizobium*, (c) *Pantoea*, (d) *Bradyrhizobium* and (e) *Pseudomonas*. For more pictures see URL link: bit.ly/3TwOgFB.

The aim of this research is to create a system that automates the process of microscopic image classification. Incorporating the computerized approach facilitates the identification process replacing or supporting human expertise and eyesight assessment with the modern computer vision image processing techniques. Machine learning methods used in this paper have already been applied to solve pattern recognition, prediction and classification problems in various fields of biology [7] and, in particular, to identify the microorganisms [8]. Some bacteria can be easily discerned from others due to their specific morphological features e.g. *Mycobacterium tuberculosis* [9] and *Escherichia Coli* [10] both having characteristic shapes. Here a fast and robust recognition scheme is in demand as these bacteria may inflict serious human illnesses. Some works perform classification not on the genera or species level but defining each class as a shape type [11]. The features relying on shape, texture or on pixel-based measures are applied in bacteria classification [12–15]. In this paper, the classification task is accomplished on the genera level via differentiating microscopic images of five selected soil bacteria genera: *Enterobacter*, *Rhizobium*, *Pantoea*, *Bradyrhizobium* and *Pseudomonas* (see Fig 1) grown in specific conditions on selected medium. Some of these bacteria genera have a positive impact on plant growth while the others are pathogenic. For this reason it is important to accurately classify their character [16, 17].

Identification of microorganisms with machine learning methods is widely applied for recognition of pathogens causing human infections (see e.g. [12]). In contrast, the topic of soil microorganism classification has not been so-far extensively investigated. In case of image-based soil microorganism identification discussed in [18], the analysis of color features used for bacteria recognition yields up to 97% of classification accuracy (ACC). In the latter work, the goal was to create a system enabling automatic recognition of samples that are preprocessed by the microbiologists. The introduced chemical reactions result in the color change of samples depending on the species of the microorganism which ultimately facilitates the efficiency of the classifier in achieving more accurate results.

In our research a different approach is adopted. The microscopic images can be taken with various microscopes and under different lighting conditions. In addition, the photographed samples can also be processed by the microbiologists upon administering a contrast or initiating a chemical reaction. Furthermore, the analyzed samples are usually colored with dyes to improve visibility of the objects examined under the microscope. In order not to rely on these factors, the different types of features based on bacteria geometry and their group dispersion are considered in this work which yields an alternative for the color-based traits classification. Developing such a set of features can help to create an automatic program that performs an accurate classification on both raw images and on those subjected to chemical reactions. The computations are performed here on images of bacteria samples that are not earlier processed by the microbiologists. In the prior research, the combination of geometric and texture features [19] calculated on the same image dataset resulted in up to 97% classification accuracy. However, in this research features based on texture are excluded as they rely on luminance (i.e. pixels intensity values which in turn may depend on lighting conditions). Instead, only features related directly to the geometry and dispersion of the analyzed objects are considered. The highest classification accuracy obtained here for such a set of features equals 85.14%. The present findings suggest that alternative feature types have the potential to supplant chrominance and luminance features in the realm of bacterial classification. Such an approach would enable classification with comparable precision for images captured under diverse illumination conditions, amalgamating preprocessed and raw images, as the outcome remains impervious to color and light influences. Notably, the investigated set of microscopic images demonstrated an accuracy of 95.6% for exemplary color features, indicating a remaining deviation of 10% in classification accuracy. Nonetheless, given the multifaceted nature of this issue, further exploration of various factors is warranted, and the classification results are anticipated to be enhanced upon adjustment of pertinent variables as explicated in the ensuing discussion section.

## Material

Strains X58AD (*Pantoea sp*), Pi72ED (*Enterobacter sp*), Ps118AA (*Pseudomonas sp*) were grown for 48 hours in 26 Celsius degrees on Plate Count Agar (BTL P-0037) medium. E77AO (*Rhizobium*) bacteria strain and a strain that was not present in Symbio-Bank (*Bradyrhizobium*) were grown on Yeast Mannitol Agar medium for 96 hours in 26 Celsius degrees. Bacteria of each strain were collected from a single colony and transferred on the surface of glass plate. In the next step, a drop of sterile water was added and mixed with the bacteria. The resulting smear was covered with microscope slide. The analyzed images were taken with a Nikon 80i microscope.

## Methods

### Work-flow scheme

The work-flow scheme applied in this work consists of the following steps:

1. Segmentation of the Region of Interest.
2. Feature Calculation.
3. Feature Selection.
4. Class Recognition.

### Segmentation of the Region of Interest

The aim of image segmentation is to separate the Region of Interest (ROI) from the background by creating a binary mask. In our case, ROI is the area where bacteria are located and this subarea of the mask is set to be white, while the background remains black. At first the image is converted to grayscale, then the Otsu method [18] combined with open and close morphological operations [20] is applied. These computations are performed with MATLAB functions: *rgb2gray*, *multithresh*, *imbinarize*, *imfill* and *bwareaopen*.

### Calculation of geometric features

The shape of bacteria depends e.g. on their genera and growth phase. The geometric features are measured here on typical bacteria instances selected from each microscopic image and applied later for the classification purposes.

#### Dependencies between vectors

Let $Q_{m}={\left\{q_{k}\right\}}_{k=0}^{m}$ be a set of *m* + 1 planar points *q*_*k*_ = (*x*_*k*_, *y*_*k*_) on a single bacteria’s boundary in 2D-Euclidean space. These points are set in a clockwise order according to the following procedure. Recall that in MATLAB function $atan2(\tilde{y},\tilde{x})$ calculates the angle between *x*-axis and a line joining point $\tilde{p}=\left(\tilde{x},\tilde{y}\right)$ with the origin of the coordinate system i.e. a point (0, 0). Upon shifting the origin to the point *c* = (*x*_*c*_, *y*_*c*_) where $x_{c}=\left(1/\left(m+1\right)\right)\sum_{k=0}^{m}x_{k}$ and $y_{c}=\left(1/\left(m+1\right)\right)\sum_{k=0}^{m}y_{k}$ we applied here *atan*2(*x*_*k*_ − *x*_*c*_, *y*_*k*_ − *y*_*c*_)—note that we also flipped variables in *atan2* to guarantee a clockwise order in $Q_{m}$. The points are thus indexed in ascending order based on the *atan2* values. We pick now a point $q_{md}\in Q_{m}$ whose Euclidean distance towards the point *c* is the smallest and then reorder points. If we have a sequence of elements *q*_0_, *q*_2_, …, *q*_*m*_ and we choose one of them as *q*_*md*_ it becomes the first element of the new sequence ${\tilde{Q}}_{m}$. Then all elements following *q*_*md*_ are shifted after *q*_*md*_, and finally we append the elements that preceded *q*_*md*_ at the end of the sequence (so if we had *q*_0_, *q*_1_, *q*_2_, *q*_3_, *q*_4_, *q*_5_, *q*_6_ and *q*_*md*_ = *q*_3_ the new order reads as *q*_3_, *q*_4_, *q*_5_, *q*_6_, *q*_0_, *q*_1_, *q*_2_). Next the set ${\tilde{Q}}_{m}$ is reduced to ${\hat{Q}}_{n}={\left\{{\hat{q}}_{i}\right\}}_{i=0}^{n}$ upon picking *n* + 1 points. In this work *n* + 1 = 10 is arbitrarily selected for all bacteria. The points forming ${\hat{Q}}_{n}$ are selected applying the following formula *fix*(*linspace*(0, *m*, *n* + 2)). Employing these functions provides a guarantee that the distances between the selected points are equal in terms of their indices, while minimizing the differences between these distances. Assume *m* is equal to 108 and *n* + 2 to 11, applying the *linspace* function results in the following values: 0, 10.8, 21.6, 32.4, 43.2, 54, 64.8, 75.6, 86.4, 97.2, 108. After processing by the *fix* function and omitting the first element, we obtain the indices of the points in ${\tilde{Q}}_{m}$—10, 21, 32, 43, 54, 64, 75, 86, 97, 108, which form the set of points ${\hat{Q}}_{n}$. In the next step we calculate distances between each ${\hat{q}}_{i}$ and ${\hat{q}}_{i+1}$ (and the distance between ${\hat{q}}_{n}$ and ${\hat{q}}_{0}$), and between each ${\hat{q}}_{i}$ and *c*.

**Fig 2:**
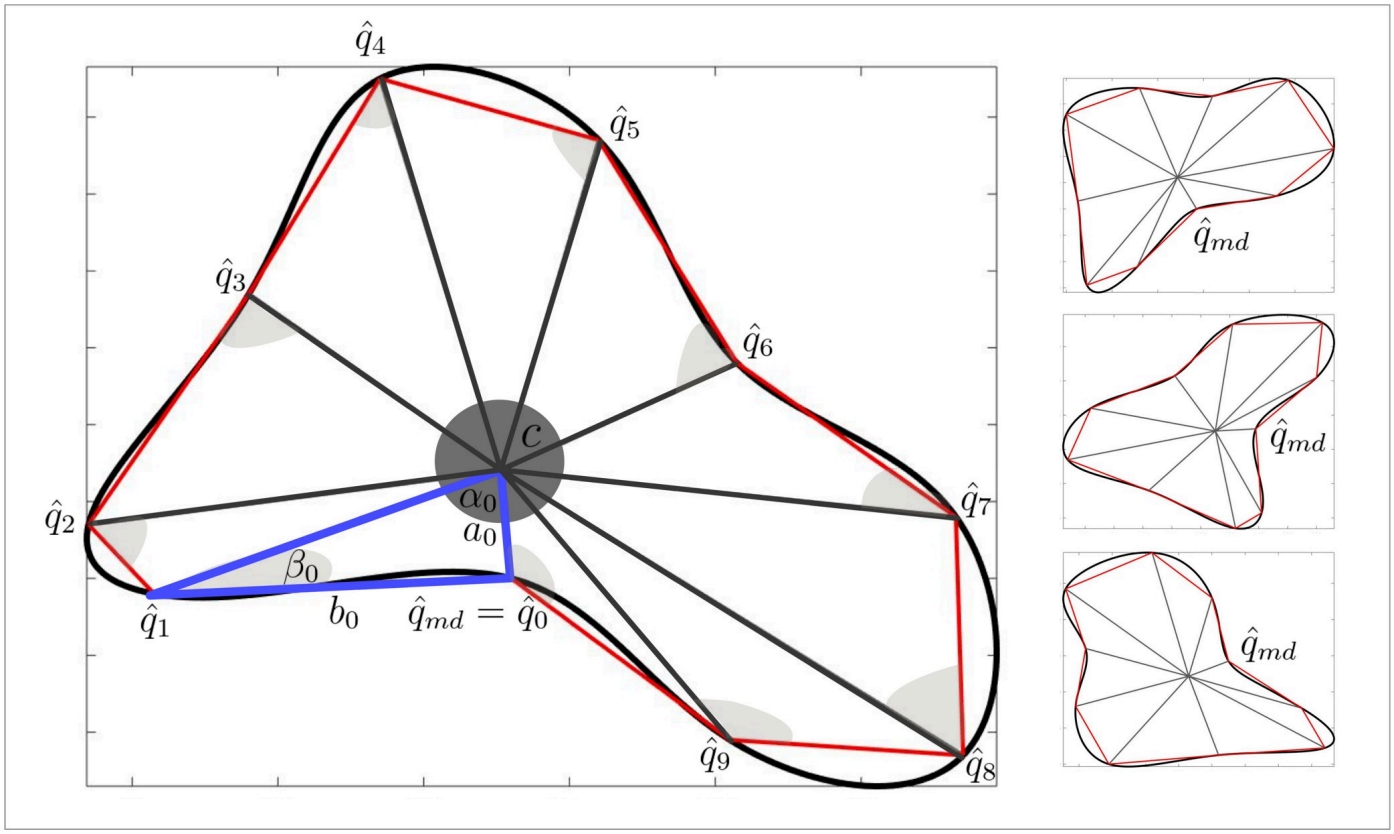
Distances and angles on exemplary shape, and exemplary rotations of a shape with applied method. Vectors: ${\vec{a}}_{k}$—dark gray lines, ${\vec{b}}_{k}$—red lines, ${\vec{\alpha }}_{k}$—dark gray angles, ${\vec{\beta }}_{k}$—light gray angles.

The latter approach is illustrated in Fig 2. Note that no matter how the figure is rotated we always pick *q*_*md*_ placed in the corresponding similar position on bacteria’s boundary resulting in a similar order of vector elements (starting with its *q*_*md*_).

In addition, for a selected *k*-th bacteria based on its boundary points ${\hat{Q}}_{n}^{\left(k\right)}$, a set of *n* + 1 triangles ${\left\{\Delta_{i}^{\left(k\right)}\right\}}_{i=0}^{n}$ is formed each determined by the vertices $\left\{{\hat{q}}_{i}^{\left(k\right)},{\hat{q}}_{i+1}^{\left(k\right)},c^{\left(k\right)}\right\}$ (the last triangle $\Delta_{n}^{\left(k\right)}$ is defined by $\left\{{\hat{q}}_{n}^{\left(k\right)},{\hat{q}}_{0}^{\left(k\right)},c^{\left(k\right)}\right\}$)—see Fig 2. Recalling that $\rho \left(x,y\right)=\sqrt{\sum_{i=0}^{n}{\left(x_{i}-y_{i}\right)}^{2}}$ defines the Euclidean distance for $x,y\in E^{n}$, define now for each ${\hat{Q}}_{n}^{\left(k\right)}$ (with ${\vec{v}}_{k,i}={\hat{q}}_{i}^{\left(k\right)}-c^{\left(k\right)}$, ${\vec{r}}_{k,i}=c^{\left(k\right)}-{\hat{q}}_{i}^{\left(k\right)}$ and ${\vec{w}}_{k,i}={\hat{q}}_{i}^{\left(k\right)}-{\hat{q}}_{i+1}^{\left(k\right)}$, for *i* = 0, …, *n*; note ${\vec{w}}_{k,n}={\hat{q}}_{0}^{\left(k\right)}-{\hat{q}}_{n}^{\left(k\right)}$) the following measures (see Fig 2):

-
${\vec{a}}_{k}$ —vector of *n* + 1 distances $\rho ({\hat{q}}_{i}^{\left(k\right)},c^{\left(k\right)})$,
-
${\vec{b}}_{k}$ —vector of *n* + 1 distances $\rho ({\hat{q}}_{i}^{\left(k\right)},{\hat{q}}_{i+1}^{\left(k\right)})$ (with $b_{k,n}=\rho ({\hat{q}}_{n}^{\left(k\right)},{\hat{q}}_{0}^{\left(k\right)}$)),
-
${\vec{\alpha }}_{k}$ —vector of of *n* + 1 angles $\text{arccos}({\vec{v}}_{k,i+1},{\vec{v}}_{k,i})$ (with $\alpha_{k,n}=\text{arccos}\left({\vec{v}}_{k,0},{\vec{v}}_{k,n}\right)$),
-
${\vec{\beta }}_{k}$ —vector of *n* + 1 angles $\text{arccos}({\vec{r}}_{k,i+1},{\vec{w}}_{k,i})$ (with $\beta_{k,n}=\text{arccos}\left({\vec{r}}_{k,0},{\vec{w}}_{k,n}\right)$).

In one microscopic image there might be hundreds of bacteria instances. Some of them are grouped together with overlaps which results in being identified as a single object once Region of Interest mask is applied. Another impeding factor comes from the fact that bacteria image can be taken at various stage of growth potentially related to its varying shape. Burying in mind the above concerns, only several *b* bacteria instances from the ROI mask are considered. These bacteria are selected based on the area value of the objects. All objects are sorted in ascending order and *b* items are selected with the area value closest to the median of all area values of the objects in a single image. Such approach ensures selection of objects representing single bacteria cells rather than groups of overlapped cells. In this research, for the calculation of features {1} and {2} we set *b* = 50 and for {3}, {4}, {5} and {6} *b* = 10 (for enumeration of features see subsection—All geometric features).

Each of the selected *b* bacteria on a given image represented by vector measures ($F_{b}={\left\{F_{k}\right\}}_{k=1}^{b}$ where $F_{k}=\left({\vec{a}}_{k},{\vec{b}}_{k},{\vec{\alpha }}_{k},{\vec{\beta }}_{k}\right)\in R^{4(n+1)}$) is compared with the exemplary bacteria measure selected by experts which is represented by $F_{e}=\left({\vec{a}}_{e},{\vec{b}}_{e},{\vec{\alpha }}_{e},{\vec{\beta }}_{e}\right)\in R^{4(n+1)}$.

**Fig 3:**
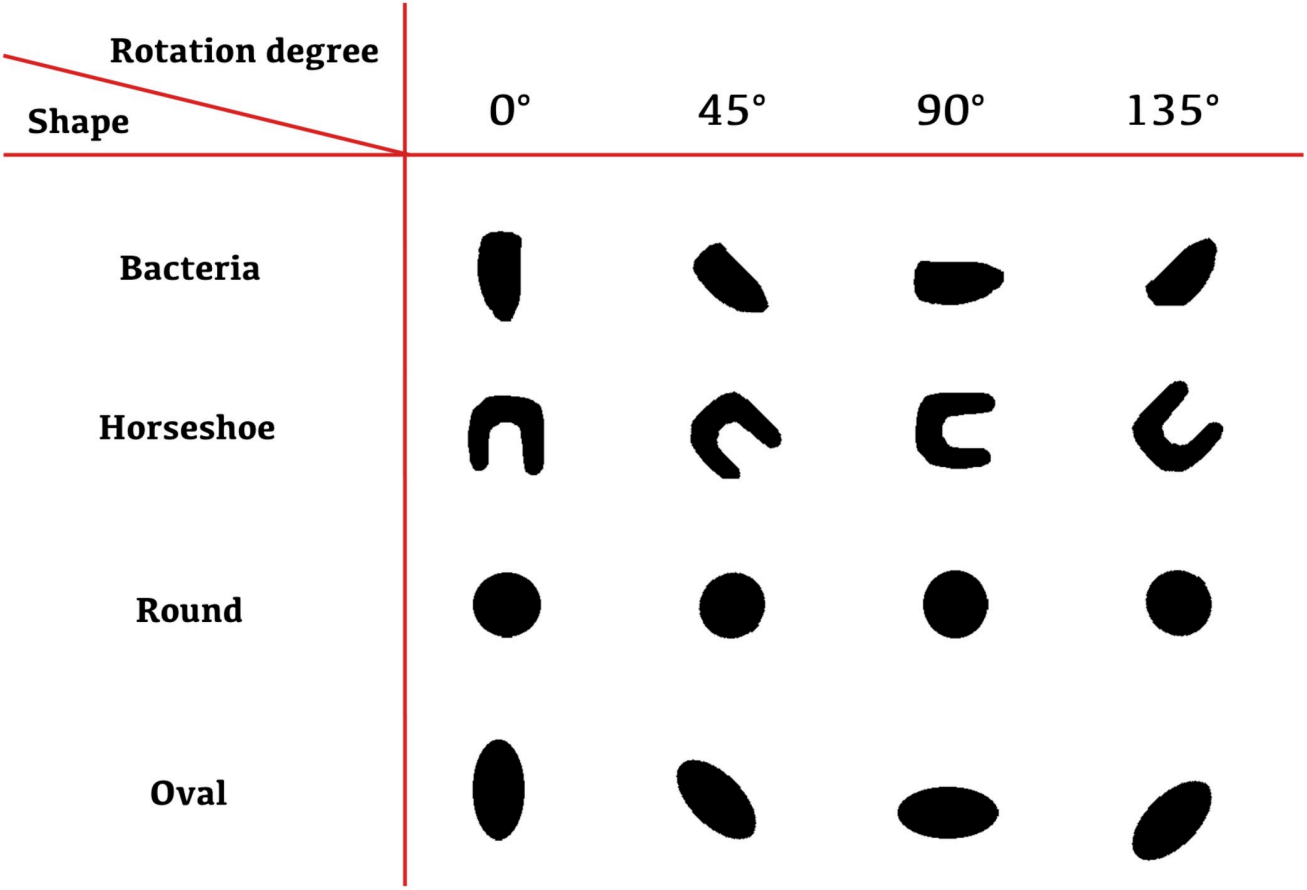
Shapes that were compared with a bacteria-like shape.

To illustrate the vector comparison procedure and to prove its credibility on more distinctive shapes an example of shape comparison between $F_{bs}=\left({\vec{a}}_{bs},{\vec{b}}_{bs},{\vec{\alpha }}_{bs},{\vec{\beta }}_{bs}\right)\in R^{4(n+1)}$ with other vectors is presented (see Fig 3). *F*_*bs*_ is a set of vectors of values calculated for bacteria-shaped object. This object is an irregular oval shape that represents a bacteria cell. *F*_*bs*_ is compared with: *F*_*b*2_—vectors calculated for bacteria-shaped object with double magnified size, *F*_*h*_—vectors calculated for horseshoe shape, *F*_*r*_—vectors calculated for a round shape and lastly, *F*_*o*_—vectors calculated for oval shape. All shapes in question are artificially created with a slight irregularity applied. The latter corresponds to the objects selected by the ROI mask as they are also irregular and not the symmetric round or oval shapes.

For a given bacteria-like shape represented by *F*_*bs*_ we calculate the Pearson coefficient value [21] for all corresponding pairs of vectors in (*F*_*bs*_, *F*_*n*_), where *F*_*n*_ ∈ {*F*_*bs*_, *F*_*b*2_, *F*_*h*_, *F*_*r*_, *F*_*o*_}, to verify how its value corresponds to the object shape Fig 3. Table 1 shows the correlation values between bacteria-shaped object and the same object resized, whereas Table 2 reports on correlation between bacteria-shaped object and other shapes. These calculations are conducted on 200 × 200 pixel images rotated by angles 0°, 45°, 90° and 135° in a counterclockwise direction around the center of the image. In this experiment, all *F*_*n*_ are calculated for each of the four selected angles yielding: $F_{n^{0^{\circ }}},F_{n^{45^{\circ }}},F_{n^{90^{\circ }}},F_{n^{135^{\circ }}}$ (e.g. for round shape we have $F_{r^{0^{\circ }}},F_{r^{45^{\circ }}},F_{r^{90^{\circ }}},F_{r^{135^{\circ }}}$). Note that *F*_*n*_ is equal to $F_{n^{0^{\circ }}}$. Calculated data show significant impact of the object shape on the coefficient value for vectors of *a* and *α*, and that coefficient value is almost independent from the object size and rotation.

**Table 1:** Table presents Pearson coefficient between vectors calculated on bacteria-shaped object *F*_*bs*_ and the same object rotated ($F_{bs^{0^{\circ }}},F_{bs^{45^{\circ }}},F_{bs^{90^{\circ }}},F_{bs^{135^{\circ }}}$) and *F*_*bs*_ with vectors calculated for bacteria-shaped object twice magnified rotated ($F_{{b2}^{0^{\circ }}},F_{{b2}^{45^{\circ }}},F_{{b2}^{90^{\circ }}},F_{{b2}^{135^{\circ }}}$).

SV	$F_{bs^{0^{\circ }}}$	$F_{bs^{45^{\circ }}}$	$F_{bs^{90^{\circ }}}$	$F_{bs^{135^{\circ }}}$	$F_{b2^{0^{\circ }}}$	$F_{b2^{45^{\circ }}}$	$F_{b2^{90^{\circ }}}$	$F_{b2^{135^{\circ }}}$
$\vec{a}$	**1.00**	**0.98**	**1.00**	**0.98**	**0.99**	**0.98**	**0.99**	**0.98**
$\vec{b}$	1.00	0.30	1.00	0.30	0.03	0.36	0.03	0.32
$\vec{\alpha }$	**1.00**	**0.97**	**1.00**	**0.97**	**0.99**	**0.97**	**0.99**	**0.97**
$\vec{\beta }$	1.00	0.92	1.00	0.92	0.97	0.93	0.97	0.93

SV stands here for the Set of Vectors which is a set that consists of vectors corresponding to $\vec{a}$, $\vec{b}$, $\vec{\alpha }$ and $\vec{\beta }$ that are compared with the corresponding vectors in *F*_*bs*_ with the Pearson Coefficient.

**Table 2:** Table presents pearson coefficient between vectors calculated on bacteria-shaped object *F*_*bs*_ with vectors calculated for horseshoe *F*_*h*_, round *F*_*r*_ and oval *F*_*o*_ shapes rotated by 0°, 45°, 90° and 135°.

SV	$F_{h^{0^{\circ }}}$	$F_{h^{45^{\circ }}}$	$F_{h^{90^{\circ }}}$	$F_{h^{135^{\circ }}}$	$F_{r^{0^{\circ }}}$	$F_{r^{45^{\circ }}}$	$F_{r^{90^{\circ }}}$	$F_{r^{135^{\circ }}}$	$F_{o^{0^{\circ }}}$	$F_{o^{45^{\circ }}}$	$F_{o^{90^{\circ }}}$	$F_{o^{135^{\circ }}}$
$\vec{a}$	**0.25**	**0.23**	**0.25**	**0.23**	**0.51**	**0.56**	**0.51**	**0.56**	**0.97**	**0.98**	**0.97**	**0.98**
$\vec{b}$	0.28	0.33	0.28	0.33	0.03	0.05	0.03	0.05	0.29	0.23	0.29	0.23
$\vec{\alpha }$	**0.07**	**0.16**	**0.07**	**0.16**	**0.46**	**0.60**	**0.46**	**0.60**	**0.91**	**0.92**	**0.91**	**0.92**
$\vec{\beta }$	0.19	0.19	0.19	0.19	0.20	0.35	0.20	0.35	0.88	0.90	0.88	0.90

SV stands here for the Set of Vectors which is a set that consists of vectors corresponding to $\vec{a}$, $\vec{b}$, $\vec{\alpha }$ and $\vec{\beta }$ that are compared with the corresponding vectors in *F*_*bs*_ with the Pearson Coefficient.

Despite the fact that Pearson coefficient properly reflects the relationship between shapes (expressed in vector forms and compared respectively), in the case of bacteria comparison, better classification results can be obtained upon replacing this coefficient with a slightly different approach presented in the following example.

To compare two vectors (e.g. representing some abstract feature), assume that the similarity between two vectors *w*_1_ = [1, 2, 3, 4] and *w*_0_ = [4, 1, 2, 3] is to be established. In doing so, the normalization of both vectors renders *w*_1*n*_ = [0, 0.33, 0.66, 1] and *w*_0*n*_ = [1, 0, 0.33, 0.66]. Then three more vectors are created as they correspond to different positions of the object (on which *w*_1_ was calculated): *w*_2*n*_ = [1, 0, 0.33, 0.66], *w*_3*n*_ = [0.66, 1, 0, 0.33] and *w*_4*n*_ = [0.33, 0.66, 1, 0]. In the next step, we calculate mean squared error [22] between each *w*_*i*_ (for *i* = 1, …, 4) and *w*_0_ which is equal to: 1.33, 0, 1.33 and 1.78, respectively. Then the smallest value is chosen, which here reads as 0 meaning that both *w*_0*n*_ and *w*_*in*_ are equal.

Vector *w*_1_ can represent a certain vector calculated on currently analyzed shape (e.g. *a*_*n*_ from *F*_*n*_) and *w*_0_ stands for a corresponding vector calculated on a bacteria-like shape (e.g. *a*_*bs*_ from *F*_*bs*_). Here all the shapes from Fig 3 are compared with the bacteria-like shape. Vectors *w*_1_ and *w*_0_ can also represent a vector calculated on currently analyzed bacteria (e.g. *a*_*k*_ from *F*_*k*_) and on the exemplary one (e.g. *a*_*e*_ from *F*_*e*_). These dependencies are calculated for every selected bacteria in the analyzed image.

Subsequently, the minimum mean square error value is computed for specific vectors corresponding to each of the *b* chosen bacteria in the image, across all four vectors. The corresponding results are denoted by $MSE{\vec{a}}_{k}^{min},MSE{\vec{b}}_{k}^{min},MSE{\vec{\alpha }}_{k}^{min},MSE{\vec{\beta }}_{k}^{min}$, where *k* = 1, 2, …, *b*. Then respective mean values of the minimum values for vectors $\vec{a},\vec{b},\vec{\alpha },\vec{\beta }$ are calculated for all the selected bacteria from an analyzed image rendering four features based on geometry: $\bar{MSE{\vec{a}}^{min}},\bar{MSE{\vec{b}}^{min}},\bar{MSE{\vec{\alpha }}^{min}},\bar{MSE{\vec{\beta }}^{min}}$.

#### Curvature and arc-length

Having selected ${\hat{Q}}_{n}$ points (described in previous subsection) one can estimate the object’s boundary with the aid of interpolation [23]. In order to define any interpolant *γ* which graph forms a closed curve the set ${\hat{Q}}_{n}$ is augmented with an extra point ${\hat{q}}_{n+1}={\hat{q}}_{0}$. The missing interpolation knots ${\left\{{\hat{t}}_{i}\right\}}_{i=0}^{n+1}$ for which ${\hat{q}}_{i}=\gamma \left({\hat{t}}_{i}\right)$ are estimated from *exponential parameterization* [24, 25]:

$$\begin{array}{c} {\hat{t}}_{i}=0,\;{\hat{t}}_{i+1}={\hat{t}}_{i}+{\left\|q_{i+1}-q_{i}\right\|}^{\lambda },\;i=0,\cdots ,n \end{array}$$

with λ ∈ [0, 1]. Here a special case of λ = 0.5 (the so-called *centripetal parameterization*) is used. Next a cubic spline $\gamma ={\hat{\gamma }}^{cs}$ with clamped boundary conditions [26] is applied (a complete spline). The latter requires an a priori information on ${\hat{\gamma }}^{\prime }\left({\hat{t}}_{0}\right)=v_{0}$ and ${\hat{\gamma }}^{\prime }\left({\hat{t}}_{n+1}\right)=v_{n+1}$ which is originally unavailable. In order to extract somehow *v*_0_ and *v*_*n*+1_ an approach based on Modified Hermite scheme is used [27], where both *v*_0_ and *v*_*n*+1_ are estimated from Lagrange Cubics ${\hat{\gamma }}_{0}^{C}$, ${\hat{\gamma }}_{n-2}^{C}$ fitting $\left\{{\hat{q}}_{0},{\hat{q}}_{1},{\hat{q}}_{2},{\hat{q}}_{3}\right\}$ and $\left\{{\hat{q}}_{n-2},{\hat{q}}_{n-1},{\hat{q}}_{n},{\hat{q}}_{n+1}\right\}$ yielding $v_{0}={\hat{\gamma }}_{0}^{C^{\prime }}\left({\hat{t}}_{0}\right)$ and $v_{n+1}={\hat{\gamma }}_{n-2}^{C^{\prime }}\left({\hat{t}}_{n+1}\right)$, respectively.

Having constructed a complete spline $\gamma ={\hat{\gamma }}^{cs}$ one can compute its curvature:

$$\begin{array}{c} \kappa \left(t\right)=\frac{\left\|{\vec{T}}^{\prime }\left(t\right)\right\|}{\left\|\vec{r}{\;}^{\prime }\left(t\right)\right\|}, \end{array}$$

where $\vec{r}\left(t\right)=\dot{\gamma }\left(t\right)$ is a tangent vector to *γ* at *t* with its normalized vector $\vec{T}\left(t\right)=\vec{r}\left(t\right)/\left\|\vec{r}\left(t\right)\right\|$ or arc-length of the curve *γ* on interval [*a*, *t*]:

$$\begin{array}{c} s=\int_{a}^{t}\|\vec{r}{\;}^{\prime }\left(u\right)\|\;du. \end{array}$$

#### All geometric features

The final set of features based on size and geometry of the selected objects reads as:

- {1} *Mean bacteria arc-length*—which is a sum of all *n* + 1 arc-lengths representing the perimeters of all selected bacteria divided by *b*,
- {2} *Mean curvature of b bacteria in one image*—a sum of all integrals of a curvature *κ*(*t*) on each of the [*t*_*i*_, *t*_*i*+1_] ∋ *t* intervals calculated for each bacteria where *i* = 0, 1, …, *n*. Then the sum of integrals is divided by *b*,
- {3} *Minimal mean square error first distance*—$\bar{MSE{\vec{a}}^{min}}=\left(1/b\right)\sum_{k=1}^{b}MSE{\vec{a}}_{k}^{min}$,
- {4} *Minimal mean square error second distance*—$\bar{MSE{\vec{b}}^{min}}=\left(1/b\right)\sum_{k=1}^{b}MSE{\vec{b}}_{k}^{min}$,
- {5} *Minimal mean square error first angle*—$\bar{MSE{\vec{\alpha }}^{min}}=\left(1/b\right)\sum_{k=1}^{b}MSE{\vec{\alpha }}_{k}^{min}$,
- {6} *Minimal mean square error second angle*—$\bar{MSE{\vec{\beta }}^{min}}=\left(1/b\right)\sum_{k=1}^{b}MSE{\vec{\beta }}_{k}^{min}$,
- {7} *Median of the object area in the image*,
- {8} *Percent of the bacteria area in the image*,
- {9} *Amount of objects in the image*—calculated sum of objects within the ROI mask,
- {10} *Amount of bacteria in the image*—calculated sum of the areas of objects within a ROI mask divided by the median of the object size in the current image.

### Calculation of dispersion features

The dataset analyzed in this research consists of the images with bacteria monocultures. Each soil bacteria genera has a different colony dispersion. For some genera the bacteria cells are located close to each other in a non-uniform fashion, whereas the others are equally distributed. This section outlines the possible tools which measure the impact of such irregularities on classification in terms of mean shift [28], *k*-means [29] and regression [30].

#### Mean shift

**Fig 4:**
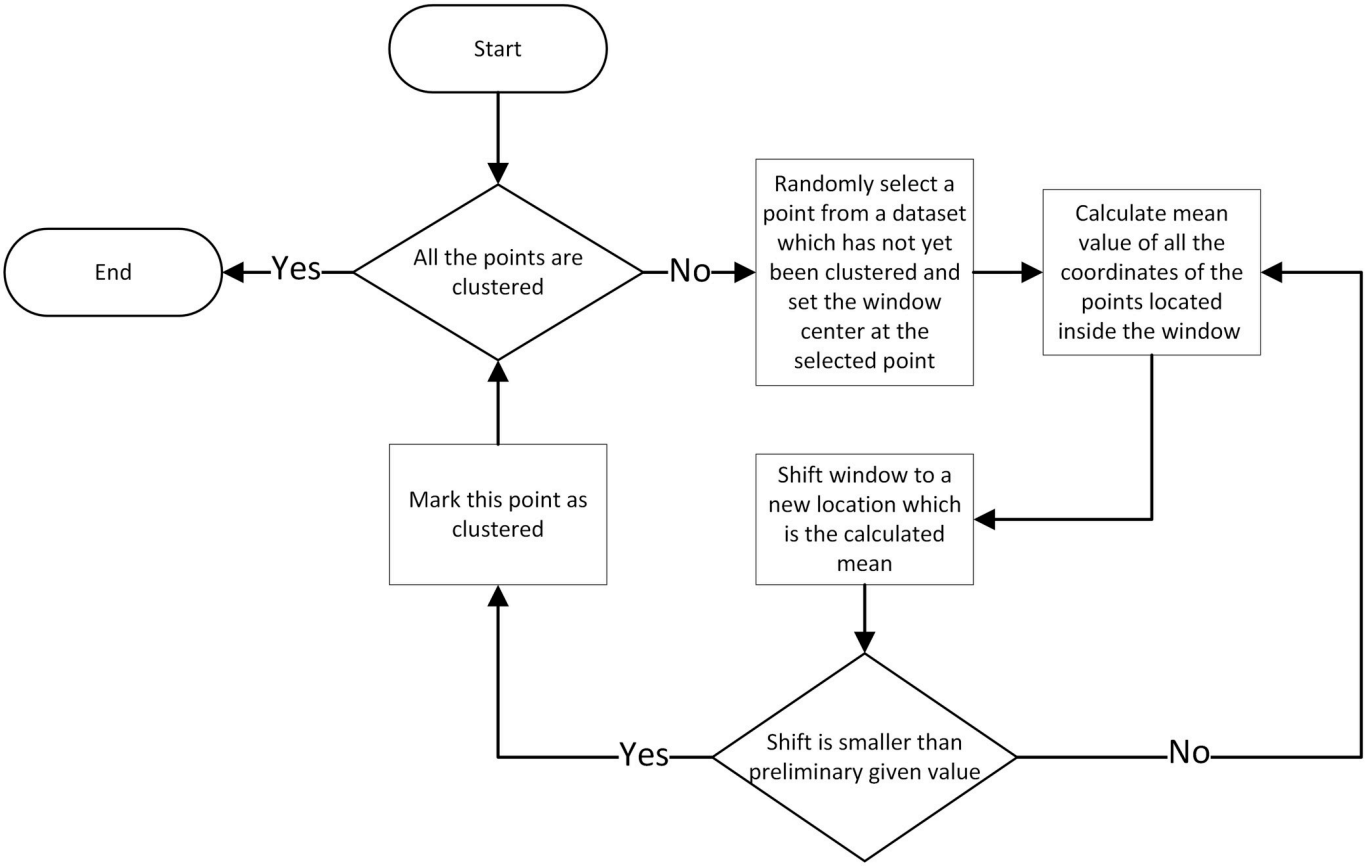
Flowchart of the mean shift algorithm.

Mean shift [28] is a scheme that allocates points through an iterative procedure to their average in a specified neighborhood (the local maxima of a density function) [31]. The output of this method consists of sets of points assigned to disjoint clusters determined by the distribution of input points. The resulting number of clusters in a clustering algorithm is determined by the algorithm itself. However, there are several input parameters that can be adjusted to customize the clustering process. These parameters include the window size, the distance metric used to evaluate the proximity of points to the cluster center and the stopping criteria for the algorithm. The mean shift algorithm flowchart is illustrated in Fig 4.

It is assumed here that the input data points of the mean shift algorithm are the centroids of the objects on the ROI mask captured with *props* MATLAB function. The generated features are the numbers of clusters to which the points were attached applying different values of *r* which is the radius of the window. The implementation of mean shift algorithm applied in this research can be found in MathWorks [32].

#### K-means

**Fig 5:**
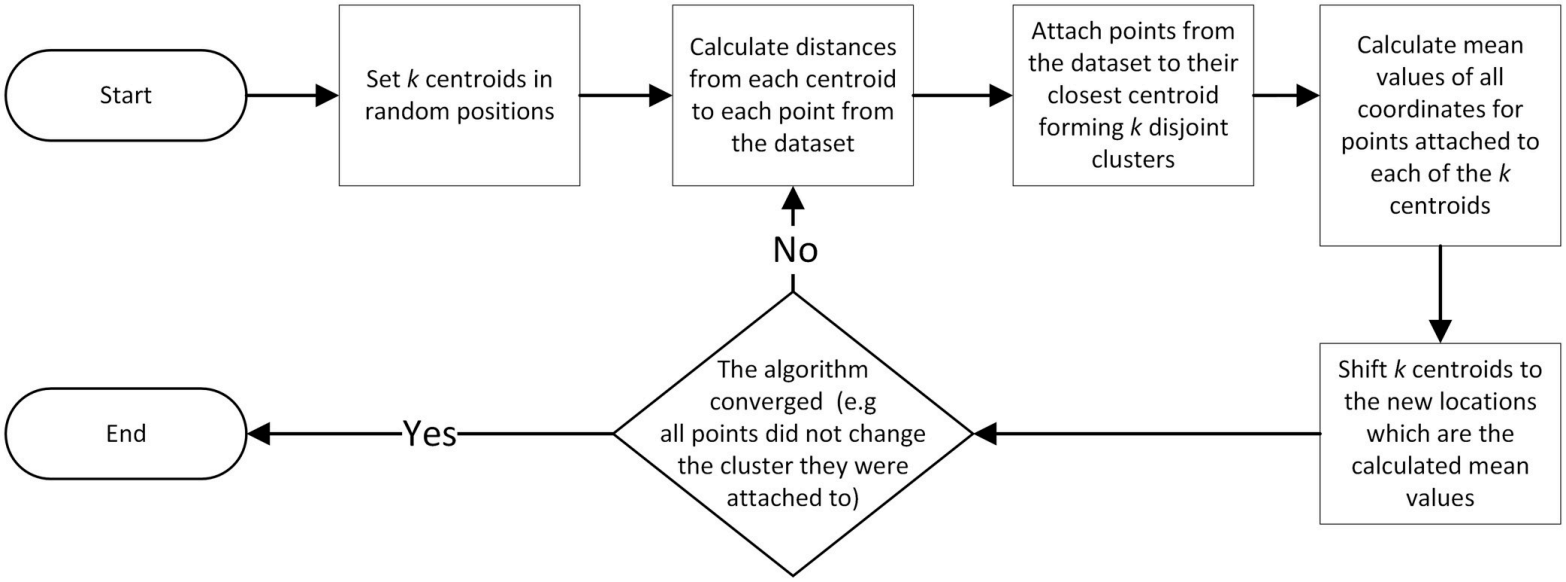
Flowchart of the K-means algorithm.

K-means [29] is a method that assigns points into *k* clusters. The algorithm is an iterative procedure of calculating distances between points and centroids, and shifting the centroids to new locations. The value of *k* is set arbitrarily. The flowchart of this algorithm is presented in Fig 5.

In order to determine features based on dispersion *k*-means method is firstly applied to cluster bacteria centroids. The latter incorporates their location in (*x*, *y*) coordinate system or both the Cartesian location combined with the area of the bacteria cell represented by (*x*, *y*, *s*). Assume the points $P_{z_{j}}={\left\{p_{i}\right\}}_{i=0}^{z_{j}}$ are given, where *z*_*j*_ + 1 defines the amount of points associated with the centroid *c*. Then a linear regression line is fitted to all points from $P_{z_{j}}$. Let $Q_{z_{j}}={\left\{q_{i}\right\}}_{i=0}^{z_{j}}$ be the points on the fitted linear regression line such that $q_{i}=\left(x_{q_{i}},y_{q_{i}}\right)$ and each $x_{q_{i}}=x_{i}$ for *x*_*i*_ being first coordinate of the point $p_{i}\in P_{z_{j}}$. Next ${\bar{d}}_{j}=\left(1/\left(z_{j}+1\right)\right)\sum_{i=0}^{z_{j}}\|y_{i}-y_{q_{i}}\|$, which is the mean distance between each of the corresponding points *p*_*i*_ and *q*_*i*_ for *j*’th centroid, is calculated. Note that ${\bar{d}}_{j}$ can also be weighted by the values of the normalized vector of bacteria surface areas *s*_*i*_ computed as ${\bar{d}}_{j}=\left(1/\sum_{i=0}^{z_{j}}s_{i}\right)\sum_{i=0}^{z_{j}}\|y_{i}-y_{q_{i}}\|s_{i}$. Such procedure is repeated for each of the *k* clusters. The resulting sum $\bar{D}=\sum_{j=1}^{k}{\bar{d}}_{j}$ becomes the feature value for currently analyzed image.

**Fig 6:**
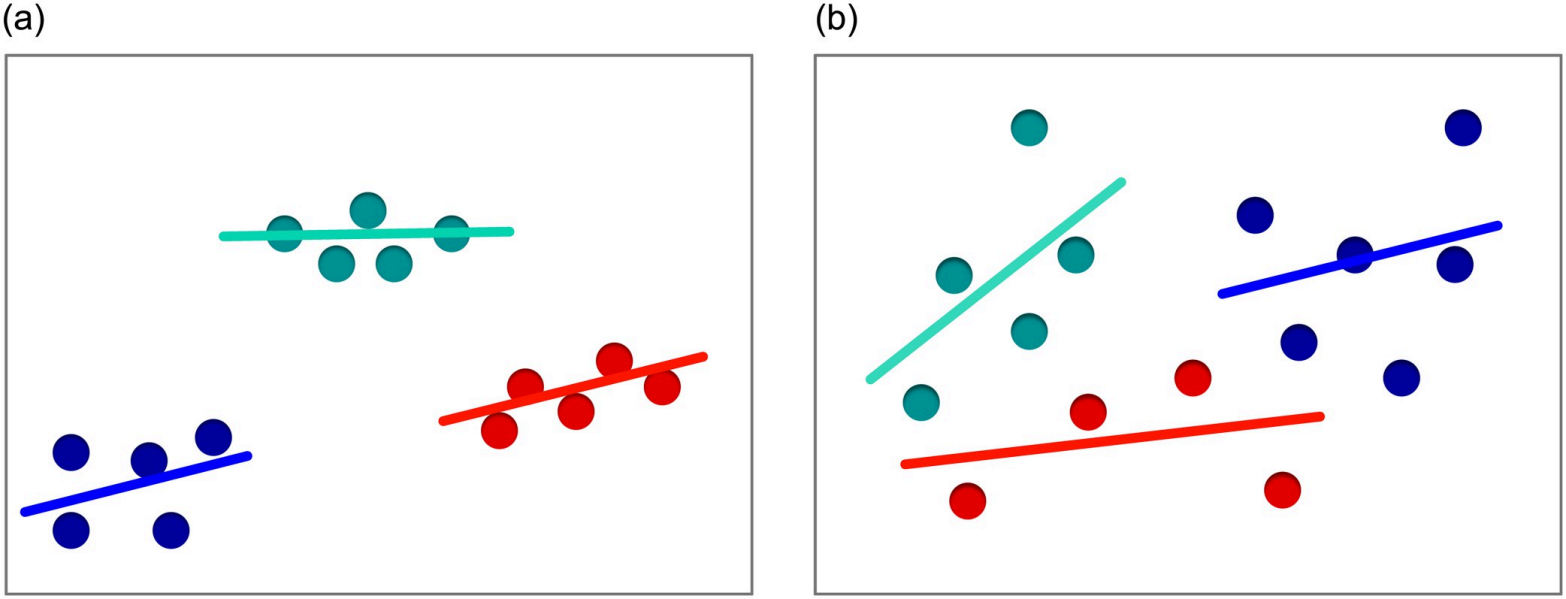
K-means algorithm and linear regression image with m + 1 = 15 for k = 3 put in sets (a) and evenly spaced (b).

**Fig 7:**
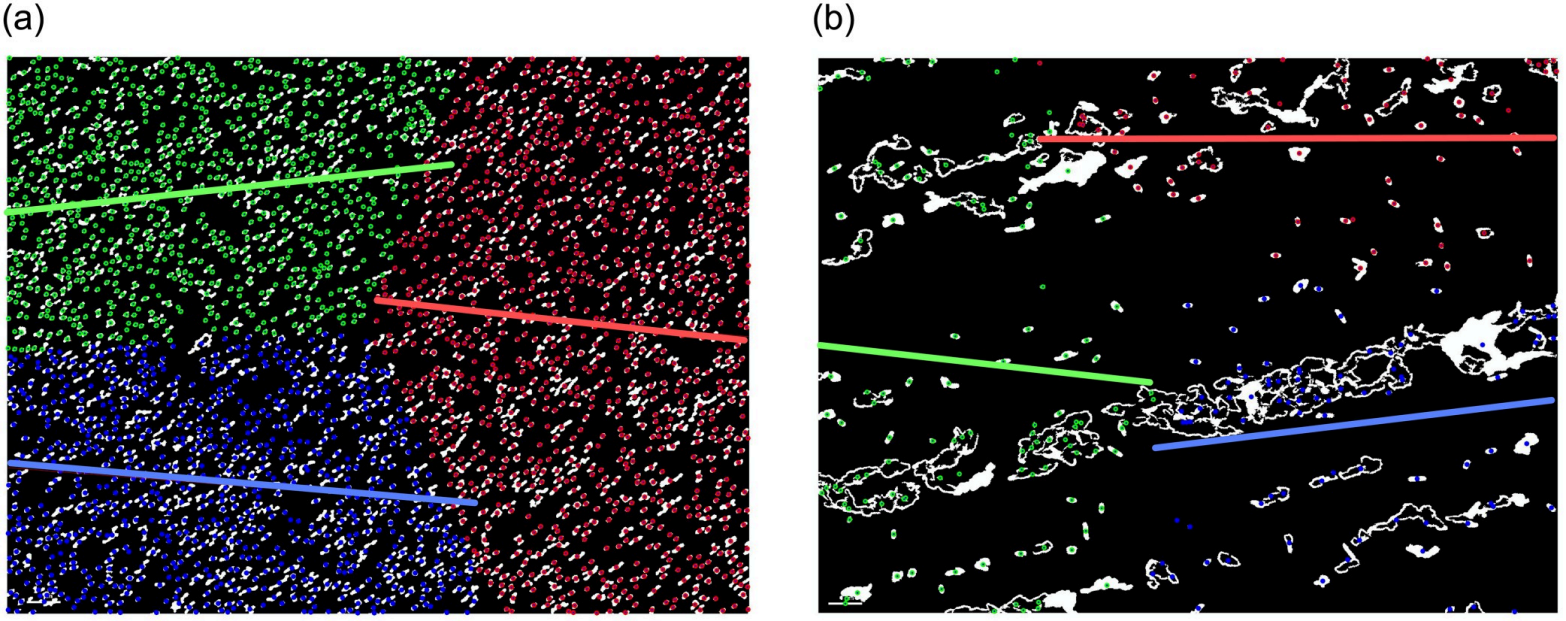
K-means algorithm and linear regression for microscopic image of *Rhizobium* (a) and *Enterobacter* (b).

To provide a clear example, consider two sets of *m* + 1 = 15 points. The first set is composed of points grouped into three subsets, while the second set contains evenly spaced samples. The points in both sets are attached to *k* = 3 clusters by *k*-means algorithm. Next, one calculates the values of $\bar{D}$ for both datasets as specified in the preceding paragraph. The computed values of $\bar{D}$ for images from Fig 6 are equal to $\bar{D}=68$ for Fig 6a and $\bar{D}=1058$ for Fig 6b. A marked discrepancy is observed in the results depending on the level of data dispersion. Fig 7 illustrates the latter used for the exemplary microscopic images.

#### All dispersion features

The final set of features based on the size and geometry consists of:

- {11-18} *Mean shift*—for different *r* values equal to = 25, 50, 75, 100, 125, 150, 175, 200, respectively,
- {19-27} *K-means and regression*—for (*x*, *y*) with *k* = 2, 6, 10, for (*x*, *y*, *s*) with *k* = 2, 6, 10 and for (*x*, *y*) with *k* = 2, 6, 10 weighted.

### Calculation of luminance and chrominance features

In this work, the classification results obtained applying features based on geometry and dispersion is compared with an accuracy rendered by features based on chrominance and luminance. In doing so, statistical measures of the pixel values, i.e. colors defined by RGB (red, green and blue) color space on the whole image or only on the area covered with the ROI mask are computed. Such features are calculated either on the image converted to grayscale or within a selected color channel. The four statistical measures employed here to analyze the data are: variance [33], mean, kurtosis and skewness [34]. The resulting set of features based on color consists of:

- {28-35} *Variance*—calculated on mask {28}, on whole image {29}, on whole image red {30}, green {31}, blue channel {32}, calculated on mask for red {33}, green {34}, blue channel {35},
- {36-43} *Mean*—calculated on mask {36}, on whole image {37}, on whole image red {38}, green {39}, blue channel {40}, calculated on mask for red {41}, green {42}, blue channel {43},
- {44-51} *Kurtosis*—calculated on whole image {44}, on the mask {45}, on whole image red {46}, green {47}, blue channel {48}, on the mask for red {49}, green {50}, blue channel {51},
- {52-59} *Skewness*—calculated on whole image {52}, on the mask {53}, on whole image red {54}, green {55}, blue channel {56}, on the mask for red {57}, green {58}, blue channel {59}.

### Feature selection

Noticeably, not all calculated features are appropriate for the classification. Some of them are not highly correlated with the affiliation to the class or their correlation with other features is too high which might cause redundancy. Such features should not be considered in the stage of class recognition. The feature selection methods solve this problem by picking appropriate features. In this work, we decided to verify the results given by the following methods:

- Fast Correlation Based Filter (FCBF) [35],
- Sparse Multinomial Logistic Regression with Bayesian Regularization (SBMLR) [36],
- Correlation-based Feature Selection (CFS) [37].

### Class recognition

Class recognition methods are used here to assign input images to certain classes representing different bacteria genera. These methods are trained on the training set and their classification performance is measured upon applying the testing set. Such sets contain selected features calculated for each of the images. Class recognition methods considered here include:

- Support Vector Machine (SVM) [38],
- Random Forest (RF) [39],
- K-Nearest Neighbors (KNN) [40],
- Multi-Layer Perceptron (MLP) [41].

These methods representing classical machine learning techniques rely on admitting features a priori determined by hand. Such class recognition methods continue to be widely used across a diverse range of applications [42]. In particular, these AI tools are also studied in the context of soil microorganism classification with high accuracy results reported [18].

#### Random forest

Random forest [39] is a group learning method whose task is to generate a set of models—trees, and then to classify the tested object into one of the classes taking into consideration the results from individual models. The trees are built based on the features table with known class assignment (supervised learning). Each node of the tree has conditions for numeric or non-numeric data. Satisfying these conditions determines object affiliation to one of the classes by the current model. In order to create a decision tree for RF (based on a table of features) one firstly randomly selects a subset of samples (table rows) with repetitions and places them into a so-called bootstrap dataset (it has as many rows as the input table of features) [43]. Having created the new dataset we draw from it *x* features (table columns) and verify which one will be the best for building the model (correctly separates the samples). The decision on which of the *x* features is to be used at a given tree node is made on the basis of methods such as e.g. Gini Impurity or Entropy [43]. The same measures allow us to set a threshold for condition concerning numeric data for a given feature. For the classification purposes hundreds of trees are generated. Upon creation of *n* trees one verifies to which class a new instance is assigned by each of the models. The final decision on the classification is made according to the majority voting rule. The effectiveness of this method is examined by comparing the achieved affiliation to a class by means of the algorithm with the actual instance assignment. One can arbitrarily select the value of *n*, however, with *n* increasing, the computational complexity of the algorithm explodes, resulting in a longer computation time. In this work the *TreeBagger* MATLAB function was applied.

## Results

The dataset considered here [44] consists of 128 microscopic images of soil bacteria from the five selected genera: *Enterobacter*—22 images, *Rhizobium*—25 images, *Pantoea*—26 images, *Bradyrhizobium*—34 images and *Pseudomonas*—21 images. These images have not been preprocessed either by the microbiologists (no chemical reactions conducted) or by any computerized system. In the experimental section the concept of cross validation [45] is applied. More specifically, 10% ratio cross validation is used, in which the set of images is randomly shuffled and divided into ten subsets. Next, nine of these sets are selected to form the training set on which our model learns how to distinguish input objects among different classes. The remaining set (called the testing set) is used to verify how good the result of classification is by calculating its accuracy. The model accuracy represents the amount of correctly classified bacteria images divided by the amount of the whole set of images (in the testing set). Then another of the ten sets becomes the testing set so that we have ten iterations (ten different training and testing sets) and calculate the mean accuracy value of ten iterations. The tables in this section display the mean accuracy resulting from 50 iterations of 10% cross-validation.

The final results include calculations based on iteratively selected parameters of class recognition methods which are: Support Vector Machine (with default parameters in *fitcsvm* MATLAB function), Random Forest with 200 trees, K-Nearest Neighbors with *k* = 1 and Multi-Layer Perceptron with network topology 15 − 15 − 15 trained with backpropagation algorithm based on gradient descent. The parameters were selected to maximize the ACC.

The accuracy for the whole set of features consisting of geometry and dispersion traits shown in Table 3 reached 85.14% for Random Forest for the whole set of five different bacteria genera. Applying feature selection methods does not increase the achieved result. The results for four different bacteria genera presented in Table 4 are also the highest for Random Forest ranging from 81.7% to 91.6%.

**Table 3:** The accuracy obtained with different feature selection and classification methods performed on features based on geometry and dispersion for the five bacteria genera.

FSM	SVM	RF (n = 200)	KNN (k = 1)	MLP	Selected features
**None**	78.3438	**85.1406**	80.5938	50.9375	{1-27}
**FCBF**	75.0312	82.2656	79.0469	63.5	{7, 11, 2, 5, 8}
**SBMLR**	40.8906	36.2344	36.2344	28.1094	{1}
**CFS**	78.3906	83.875	79.8594	60.1719	{1, 2, 5-7, 10, 11, 14}

FSM stands here for the Feature Selection Method.

**Table 4:** The accuracy computed with different classification methods performed on the whole set of features based on geometry and dispersion for four selected bacteria genera (subsets of five bacteria genera).

Selected bacteria genera	SVM	RF (n = 200)	KNN (k = 1)	MLP
**1, 2, 3, 4**	81.7944	86.2243	85.9252	58.8785
**2, 3, 4, 5**	76.1132	81.6792	80.3208	54.9057
**3, 4, 5, 1**	75.8641	84.1748	78.6408	51.9223
**4, 5, 1, 2**	88.3922	88.4314	86.8235	64.8627
**5, 1, 2, 3**	86.0638	**91.5532**	87.8298	69.617

1, 2, 3, 4 and 5 stand for the following bacteria genera respectively: *Enterobacter*, *Rhizobium*, *Pantoea*, *Bradyrhizobium* and *Pseudomonas*.

Table 5 presents accuracy for different sets and subsets of features. Features based on dispersion obtained the best accuracy of 63.72% for KNN, whereas features based on geometry reached 82.59% for Random Forest. Combining these sets increases the result by 2.55 percentage points and amounts to 85.14% for Random Forest. One can also analyze the results of the selected subsets of features based on dispersion and geometry. As an example, features extracted based on mean shift yields up to 63.94% accuracy, whereas features based on *k*-means render 46.86%. Applying features based on vectors reached only 46.86% accuracy; however, one of them—feature number 5—is accepted by both FCBF and CFS feature selection methods which is shown in Table 3 what proves its significant impact on increasing the classification accuracy.

**Table 5:** The accuracy obtained with different classification methods performed on different sets of features based on color, geometry and dispersion (and their subsets).

Set of features based on…	SVM	RF (n = 200)	KNN (k = 1)	MLP
**geometry and dispersion {1-27}**	78.3438	**85.1406**	80.5938	50.9375
**geometry {1-10}**	74.7969	**82.5938**	70.9062	53.0312
**vectors (from geometry) {3-6}**	47.5469	**48.5469**	41.9844	32.0625
**dispersion {11-27}**	60.4531	63.4219	**63.7188**	42.5312
**k-means (from dispersion) {19-27}**	39.8281	**46.8594**	43.7188	24.7976
**mean shift (from dispersion) {11-18}**	53.7188	**63.9375**	63.2812	49.9219
**color {28-59}**	86.2188	94.2969	**95.5938**	88.3906
**color, geometry and dispersion {1-59}**	89.7969	**94.8281**	91.2031	75.4688

The features calculated with *k*-means may seem insignificant. For this reason the classification results are presented depending on the amount of bacteria analyzed on a single image based on their area value. For each of the calculations, as shown in Table 6, a different quantile value is selected which means that analyzed bacteria are ones which area exceeds or is equal to that quantile area value in a single image. The 60 features were calculated for *k*-means for each of the quantiles: 0, 0.2, 0.4 and 0.6. For example for quantile equal to 0.2 the calculated features are: for k = 1, …, 20 and 2 dimensional vector for k-means, 2 dimensional vector for k-means with weighted variance and 3 dimensional vector for k-means—yielding 60 features for this quantile. The highest results are reached for the quantile equal to 0.4 amounting to 61.8%. It is remarked here that extending the final set of features by these 60 *k*-means features does not improve the final result. For that sheer reason only nine previously calculated features based on *k*-means are chosen.

**Table 6:** The accuracy with different classification methods for the five bacteria genera performed on features based on k-means and regression. The set consists of 60 features, for k = 1, …, 20 and 2 dimensional vector for k-means, 2 dimensional vector for k-means with weighted variance and 3 dimensional vector for k-means.

Quantile	SVM	RF (n = 200)	KNN (k = 1)	MLP
**0**	48.0469	58.9062	48.2031	28.2031
**0.2**	45.7812	60.625	41.875	26.5625
**0.4**	50.8594	**61.7969**	44.5312	26.0938
**0.6**	40	50.3125	36.6406	23.5938

The value in Quantile column informs that the bacteria were taken into account if their area value was greater then certain quantile of area value of the bacteria on a given image.

In this research the highest classification accuracy for a set of geometry and dispersion features yields 85.14%. In previous work [19], based on the same image data set, the accuracy obtained amounts to 97%. The latter analyses different set of features, involving geometric and texture characteristics. The texture features rely on luminance and chrominance, which may artificially improve the accuracy of the results. For example, this may occur when microscopic images from each genera are taken under different lighting conditions. Thus, the obtained accuracy 85.14% forms a promising result as the examined features are not based on color information.

## Discussion

The classification based on extracting features from bacteria geometry and dispersion yields a promising 85.14% ACC. The latter is reached for the Random Forest classifier to identify five selected soil bacteria genera. The experiments conducted on features based on geometry and dispersion separately rendered 82.59% in case of Random Forest and 63.72% for K-Nearest Neighbors. These results illustrate that applying a proper set of features with no color traits enables classification of soil bacteria. The latter permits to bypass the impact of lighting conditions and coloring of samples on classification. In contrast the geometry and dispersion based classification is insensitive to the last two factors. However, the difference between the classification accuracy based on geometry and dispersion traits versus this one based on color traits is significant (around 10 percentage points) and there are some issues requiring future research investigation.

Indeed, one needs to apply a different method of selecting points on bacteria boundary to highlight the characteristic elements of its shape. In addition, various parameterizations to estimate the unknown interpolation knots combined with different interpolation fitting schemes might also be considered [46]. Other methods for object dispersion in the image should also be examined. In this work we compared the results given by the four classification methods: Support Vector Machine, Random Forest, K-Nearest Neighbors and Multi-Layer Perceptron. Other classifiers such as Extreme Learning Machines or Deep Learning Methods may provide more effective recognition tools. The features in the future research can be also computed applying Convolutional Neural Networks [47].

The generated results are calculated on the dataset with a single bacteria genera on an input image. These organisms were grown under laboratory conditions, with no contamination involved (as they are all immersed in uniform medium). In future research, the testing should also be performed on images taken from the natural environment (e.g. from the genuine rizosphere sample). The ultimate goal is to classify different bacteria genera mixed with extra organic or non-organic objects as they cohabit in a real soil sample. More importantly, the classification results on images that contain different bacteria genera (for example mixes of two or three genera on one image) should also be examined. In particular, the final recognition tool should allow to assess the quantity of bacteria cells affiliated to a certain genera on the currently analyzed microscopic image.

The classification system created in this work can be applied in practice. However, further research is needed for samples containing strains of different species of bacteria representing the same genus. These species differ in phenotypic features (morphological, biochemical and physiological). The number of analyzed strains of bacteria has an important meaning. We are unable to draw a conclusion from a single photograph of cells or bacterial colonies known to be of some type of bacteria. As an example, the genus *Pseudomonas* includes both fluorescent and non-fluorescent bacterial species. Problems with identifying bacteria based on their morphology result from reasons such as: (i) the influence of the environment, i.e. the composition of the medium and incubation time on the cell morphology, (ii) the phase of the bacterial cell cycle, (iii) the common morphology of cells of different types of bacteria. It is worth mentioning that so far there are over 10 thousand species of culturable bacteria, with a huge number of species that cannot be cultured in the laboratory. It is very important to accurately classify the bacteria as a representative of the appropriate species. The latter permits to decide whether to use it for utilitarian purpose e.g. in biological protection of plants against diseases or to apply suitable control against a given organism if it causes diseases (pathogen) or is harmful in any other respect.
